# Evolutionary conservation of the antimicrobial function of mucus: a first defence against infection

**DOI:** 10.1038/s41522-018-0057-2

**Published:** 2018-07-04

**Authors:** Cassie R Bakshani, Ana L Morales-Garcia, Mike Althaus, Matthew D Wilcox, Jeffrey P Pearson, John C Bythell, J Grant Burgess

**Affiliations:** 1 School of Natural and Environmental Sciences, Newcastle University, Newcastle upon Tyne, UK; 2 Institute for Cell and Molecular Biosciences, Newcastle University, Newcastle upon Tyne, UK

## Abstract

Mucus layers often provide a unique and multi-functional hydrogel interface between the epithelial cells of organisms and their external environment. Mucus has exceptional properties including elasticity, changeable rheology and an ability to self-repair by re-annealing, and is therefore an ideal medium for trapping and immobilising pathogens and serving as a barrier to microbial infection. The ability to produce a functional surface mucosa was an important evolutionary step, which evolved first in the Cnidaria, which includes corals, and the Ctenophora. This allowed the exclusion of non-commensal microbes and the subsequent development of the mucus-lined digestive cavity seen in higher metazoans. The fundamental architecture of the constituent glycoprotein mucins is also evolutionarily conserved. Although an understanding of the biochemical interactions between bacteria and the mucus layer are important to the goal of developing new antimicrobial strategies, they remain relatively poorly understood. This review summarises the physicochemical properties and evolutionary importance of mucus, which make it so successful in the prevention of bacterial infection. In addition, the strategies developed by bacteria to counteract the mucus layer are also explored.

## Introduction

Bacterial infection in humans is becoming increasingly problematic, particularly with the rise of multidrug resistance in opportunistic pathogens, resulting in reduced effectiveness of antibiotics.^1^ Consequently, there is an urgent need to develop innovative approaches to tackle infection. An important strategy adopted by many living organisms to combat tissue incursion by microbes, is the secretion of a surface mucus layer.^2^ This physicochemically exceptional interface appears to have first evolved in the ctenophores^3^ and cnidarians.^4^ However despite its clear importance and widespread presence in higher organisms, the mechanisms of mucus production and its functionality are still largely unknown, except in the case of the human MUC2 gene product.^5^ A more comprehensive understanding of the chemical and physical properties of mucus might suggest novel antimicrobial strategies for clinical application.

Mucus also constitutes an essential feature of the innate immune system, considered to be universal within most phyla of both aquatic and terrestrial metazoans,^6^ and plays a pivotal role in the prevention of disease. The mucus layer functions as a protective adherent secretion coating epithelial cells which line bodily surfaces, primarily those that are routinely exposed to the external environment and therefore microbes, including potential pathogens.^7^ In humans, mucus coats the surface of the respiratory, gastrointestinal and urogenital tracts,^8^ along with the middle ear^9^ and the ocular surface and tear film,^10^ which receive mucus from the lacrimal glands.^11^ Aquatic invertebrates and fish also use a mucus layer to protect the body, gill and gut surfaces.^12^ In addition to serving as an antimicrobial barrier and a physically protective layer, mucus has several physiological functions. Importantly, mucus allows the exchange of oxygen, carbon dioxide^13^ and nutrients and metabolites, whilst lubricating surfaces, reducing damage due to shear,^14^ as well as reducing dehydration of the epithelia and providing the polymeric matrix which enables ciliary-mucus particle transport.

Although there are diverse functions of mucus, this review will focus on the physical and chemical properties of mucus responsible for its antibacterial activity and the evolutionary conservation of these features throughout metazoan development. Subsequent adaptations of microorganisms to overcome the mucus barrier, to penetrate and degrade mucus will also be discussed. The potential biomedical and biotechnological applications of mucus are also presented.

### Evolution of the mucus layer

The synthesis and secretion of a functional surface mucus layer first arose in the Cnidaria^4^ and Ctenophora.^3^ The Cnidaria are a phylum of approximately 11,000 species^15^ of predominantly marine invertebrates, which includes corals, anemones and jellyfish. This phylum can be characterised as the first phylum to have evolved radial symmetty, a medusoid or polyploid body form and stinging cells called cnidocytes.^16^ The Ctenophora are a sister-group to the Cnidaria and represent the most distant animal relatives of humans.^17^ Analogous to the Cnidaria, they are diploblastic and radially symmetrical^18^ and are known more commonly as comb jellies. Despite the investigation of Cnidarians as model systems used to study the evolution and developmental biology in metazoans, the importance of the evolution of a functional mucus layer in invertebrates is often neglected.

In corals, the luxury carbon hypothesis (LCH), proposed in the 1980s, that the primary function of mucus secretion was to remove excess photosynthetic carbon^19^ produced by symbiotic zooxanthellae. This excess results from low dietary nitrogen which restricts the allocation of carbon to growth.^20^ This theory suggests that secretion of mucus, a carbon rich compound, fulfils this need. However, it is unlikely that mucus, containing nitrogen rich glycoproteins, would have evolved for this sole purpose.^21^ It would be more appropriate, in this case, to excrete a high energy/low nitrogen compound such as a lipid or simple sugar, which is the case in aphids, for example.^22^ Furthermore, for tropical coral species where nitrogen, essential for sustaining photosynthesis in coral symbionts,^23^ is limiting,^25^ mucus production ameliorates the N-limitation problem, as it represents a greater proportion of the N-budget. In corals, the LCH is not applicable to other Cnidarians that also possess a functional surface mucosa, but which do not contain photosynthetic symbionts. Therefore, whilst mucus secretion can provide a vehicle to excrete excess carbon in symbiotic corals, this is unlikely to be the primary function of mucus secretion.

It has since been suggested that mucus first evolved in corals primarily for ciliary-mucus driven particle feeding and/or to prevent smothering by sediments, thus simultaneously providing physical protection as well as a means of accessing greater dietary nitrogen via particle capture.^24^ For example, corals are known not only to use mucus in trapping particles, but to then transport trapped particulates, bacterial cells and detritus towards the mouth and gastrovascular cavity via a process of cilia mediated entrainment.^25^ Entrainment, in this case, describes the ability of mucus to be dragged as a connected sheet or string across the epithelial surface. The processes of particle entrapment, entrainment and transport depend on the specific properties of mucus, including its polymeric glycoprotein structure, which confers high viscoelasticity and tensile strength. These properties ensure that ciliary-mucus sheet transport is efficient, which is a universal requirement in corals whether or not they possess symbionts. The selective evolution of these properties also serves an additional function—that of a physical barrier to bacteria.

Continuous mucus production and release is clearly important and characteristic of many aquatic organisms, despite the high associated energy costs. For example, the reef-building coral *Acropora acuminata* is thought to dedicate up to 40% of its daily net carbon fixation to this task alone.^25^

Although additional molecular evidence is needed, it can be postulated that the development of the mucus layer represents a major event in the evolutionary history of living organisms, one that appears just as significant as the ‘text-book’ defining characteristic of a blind gut (gastrovascular cavity) in the phylum Cnidaria. Poriferans, commonly known as sponges, are basal metazoans that precede the Cnidaria evolutionarily. There is some evidence to suggest that Poriferans possess genes and genetic structures which maybe evolutionary precursors of mucins,^3,26,27^ and in the cases of the barrel sponge *Xestospongia testudinaria* and the silvery blue sponge *Lamellodysidea herbacea* are also able to secrete some mucus.^28,29^ However, interestingly, evidence of a functional surface mucosa in Poriferans is currently lacking. Sponge tissues are continuously inundated with water and environmental bacteria, with bacterial cells occurring throughout these tissues and contributing 40–50% of their wet mass,^30^ although in some species this may be considerably lower. While ctenophores and cnidarians, in particular corals, possess a microbiota that is not only distinct from that of their immediate environment, but also other coral species,^24,31–33^ the population levels of bacteria within the tissues are much lower than in the sponges and the ‘core microbiome’ of corals appears to be relatively restricted.^34^ The evolution of effective barrier properties in a functional surface mucosa therefore appears to be associated with the general exclusion of bacteria from the bodily tissues, except for a select core microbiome. Mucus generally serves to keep eukaryotic cells and bacterial cells apart, therefore, in the absence of a separating mucus layer, bacteria are found throughout sponge tissues. With a mucus layer, as in the Cnidaria, non-commensal bacteria are essentially excluded from cnidarian tissues. It is plausible that, because sponges do not have a mucus barrier layer their tissues are more inundated with bacterial cells, and as a result, powerful antimicrobial secondary metabolites are required to keep microbial growth in check. The lack of developed tissue structures in the Porifera^35^ may be attributed to this inability to exclude bacteria from bodily tissues, and the evolution of a mucus barrier layer may have therefore been a critical evolutionary step in the development of the Ctenophora and Cnidaria. Detailed formal analysis of the evolution of mucin genes has begun, allowing a phylogeny of the gel-forming mucin like genes to be established.^3^

The exclusion of non-commensal bacteria may have initiated the evolution of the alimentary canal and therefore the evolution of higher organisms. The water-land transition and terrestrial life also required adaptations to air breathing, resulting in the evolution of respiratory surfaces (originating from the alimentary canal) which faced the problem of providing sufficient gas exchange on the one hand and being exposed to microorganisms and particulate fouling on the other.

An airway epithelium lined with mucus evolved to serve as a particle and pathogen trap, preventing microbes from penetrating and infecting gas-exchanging regions in mammalian lungs. Mucus and trapped particles are cleared from the lungs by cilia- mediated mucus entrainment—essentially the same mechanism used for feeding by corals, filter feeding ascidians and bivalves and ciliary gliding by lower invertebrates.^36^ Histological studies on lungfish (*Neoceratodus forsteri* and *Protopterus aethiopicus*), the oldest living ancestors of tetrapod vertebrates, revealed the presence of ciliated cells within the intestine.^37^ Furthermore, in *P. aethiopicus*, ciliated cells and mucus secreting cells are present in the anterior parts of the lungs,^37^ suggesting that during vertebrate evolution, cilia-mediated mucus entrainment might have been lost in the mammalian gut surface mucosa, whereas it developed into a highly efficient particle clearance system in the airways.

**Fig. 1:**
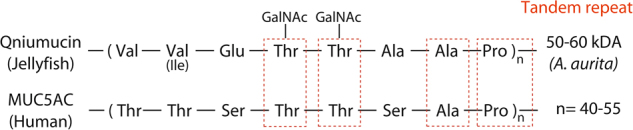
Similarity in the amino acid sequence between qniumucin from jellyfish and human MUC5AC. A similar tandem repeat of eight residues is found in both mucins and the boxes highlight four of these similarities. Although the human mucin is more flexible, both proteins form a gel in water. Reprinted (adapted) with permission from (Masuda et al. Mucin (qniumucin), a glycoprotein from jellyfish, and determination of its main chain structure. J. Nat. Prod. 70, 1089–1092 (2007). Copyright (2007) American Chemical Society

**Fig. 2:**
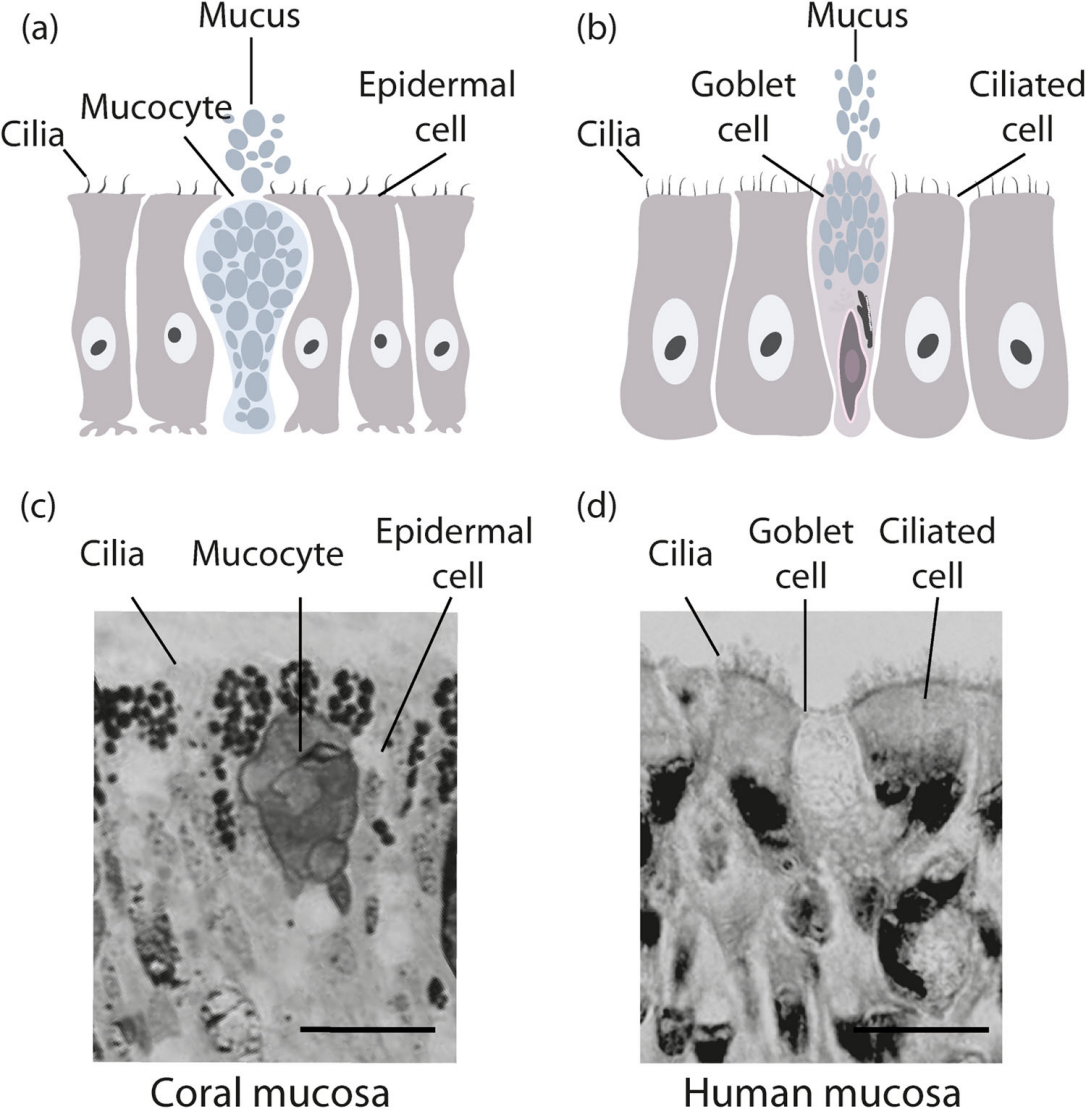
Similarity between (**a**) mucus secretory cells of Cnidarians (**b**) human airway epithelium. Coral mucocytes and human goblet cells are structurally similar and perform similar roles, which can be seen in the histological images of (**c**) a section from the coral *Coelastrea aspera*, stained with toluidine blue,^177^ showing coral mucocytes surrounded by ectodermal cells (scale bar 10 µm) and (**d**) a section of human trachea, H & E staining, showing human goblet cells surrounded by ciliated epithelial cells (scale bar 20 µm)

The evolutionary history of mucin genes is somewhat convoluted and these structurally complex glycoproteins are thought to be derived from the same ancestor as the von Willebrand Factor (vWF),^26^ a glycoprotein involved in the mediation of platelet adhesion within the blood.^38^ This is due to the occurrence of the von Willebrand D domain in both mucins and vWF. Within mucins, this domain is responsible for the polymerisation of mucin monomers through production of intermolecular disulphide bonds, subsequently allowing the gel forming mucin polymeric structure.^3^ Despite these insights, the distribution of mucin genes across all phyla is not well studied, with a concentration predominantly on human mucin genes and their evolutionary divergence from one another.^3,39^ One study that did focus on early evolution found that mucus isolated from several species of jellyfish, including *Aurelia aurita*, *Chrysaora melanogaster* and *Rhopilema esculenta* possessed a qniumucin gene which showed surprising structural similarity to MUC5AC^40^ (Fig.1), an important mucin found in the human stomach and lungs.^41^ Similarly, mucins isolated from the blue blubber jellyfish, *Catostylus mosaicus* had an amino acid content high in Thr, Ala, Pro and Glu, which is characteristic of bovine mucins.^42^ Therefore, whilst it has been identified that secreted mucins likely evolved in early metazoans and membrane-bound mucins evolved in the first vertebrates,^26^ suggesting there is some degree of divergence in their evolution, the structural and functional similarities between the mucins of early metazoans and higher mammals suggest they may be functionally similar.^40,42^ This notion is reinforced by the similarities seen in the structures of the mucus secreting cells of cnidarians and humans (Fig. 2) and also in mucus composition, which is comparable between taxonomically distinct phyla.^43^

### Mucus composition

Mucus is predominantly composed of water, which equates to approximately 95% of its overall wet mass, with the remaining 5% composed of mucin glycoproteins (~3%) and other molecules (~2%).^44,45^ The 2% constitutes both cellular debris and co-secretions of soluble proteins such as secretory IgA, peptides, lipids and nucleic acids,^46^ which fulfil specific roles.

The high water content requires the presence of regulatory mechanisms which control the hydration of mucus lined air-exposed surfaces such as the mammalian respiratory tract, allowing both appropriate supply of water into mucus and air into lungs. Secretion and absorption of water into and from the mucus is facilitated by electrolyte secretion and absorption across the underlining epithelial cells; a molecular machinery which evolved as an adaptation to terrestrial life.^47^ The importance of the hydration state of mucus and its link to transepithelial electrolyte transport is evident in the human disease cystic fibrosis, where genetic defects leading to either impaired abundance/activity of the chloride/bicarbonate secreting ion channel CFTR (cystic fibrosis transmembrane conductance regulator) or enhanced activity of the sodium absorbing ion channel ENaC (epithelial sodium channel)^48,49^ cause dehydration^50^ and high mucus viscosity.^51,52^ This prevents cilia-mediated particle clearance which eventually results in airway mucus plugging and lung infection.^52^

Mucin glycoproteins are the component of mucus that promote its function as a lubricating gel,^42^ making mucus layers non-shear resistant and shear compliant gels. While functionally conserved, mucin glycoproteins are structurally complex^43^ and separated into secreted soluble, secreted gel-forming and membrane-bound / transmembrane mucins.^5,45^ Multiple transmembrane mucins have now been identified.^7,53,54^ They are thought to be involved in cellular signalling^26^ and on the airway surface, for example, they are used to create a size exclusion barrier.^55^

Mucins can be characterised by the presence of a single mucin domain, abundant in large repeat sequences rich in proline, threonine and serine and commonly referred to as the PTS domain.^26,56^ Mucins are among the largest known macromolecules, between 200 kDa and 200 MDa in size^57^ and they contain a large number of O-linked oligosaccharide side-chains.^42,58^ These are composed of approximately 5–15 monomers that are added post-translationally, however the process is not precisely replicated, leading to variation in the size of the glycoprotein in nature. The oligosaccharides attach to the protein core via *O*-glycosidic bonds on hydroxyl side chains of both serine and threonine.^45^ There is also sparse glycosylation with branched oligosaccharides N-linked to asparagine.^59^ In particular it is the oligosaccharide chains^42^ and polymeric structure^60^ that are thought to confer the highly viscoelastic properties of mucus, which confer its barrier properties.

The oligosaccharide chains also play a crucial role in mucin storage and secretion.^61^ Intracellular gel-forming mucins are stored in a compact and condensed form in granules within mucus-secreting cells. The condensed storage of mucins containing anionic oligosaccharide chains is possible due to a high concentration of calcium ions and protons within the granules, which both mask the negative oligosaccharide charges and prevent electrostatic repulsion and thus expansion of the mucin molecules.^62,63^ In addition, non-covalent interactions take place between the D domains. Upon secretion of these granules, the mucins expand 1000–3000 fold, taking up water to form a gel, as calcium is exchanged for sodium and the pH rises.^64^ The presence of bicarbonate significantly accelerates the uptake of calcium, forming calcium carbonate and calcium bicarbonate,^61^ which is present within the surface liquid. In addition to water, bicarbonate is therefore an important determinant of mucus secretion, hydration and transportability.^61^ In cystic fibrosis, for example, functional absence of CFTR reduces the bicarbonate concentration within the airway surface liquid, meaning secreted mucins are more condensed and viscous.^65^ The integral roles of both water and bicarbonate highlight the importance of the secretory properties of mucosal surface epithelia.

### Mechanisms of the mucus barrier function

#### Physical mechanisms

Within terrestrial organisms, the important functions of mucus are to lubricate and hydrate the epithelium^66^ as well as to provide protection from bacterial infection. The antibacterial properties of mucus are multifaceted, with some of them occurring as a consequence of its other roles, it is difficult to assign primary or secondary functions of the mucus layer, as they are all intrinsically interlinked. Mucus prevents the passage of bacteria due to its unique physicochemical characteristics. For instance, its high viscosity prevents the penetration by pathogens, whilst remaining permeable to water, gases and odorants.^67^ The physicochemical properties of mucus and therefore its ability to trap particles, may also be directly correlated to the associated phospholipid and glycolipid content, as removal of these lipids can reduce viscosity by up to 85%.^68^

In addition to their gel structure, mucus layers are continually removed and regenerated,^69^ which detaches contaminants rapidly and efficiently before they can reach the underlying surface tissues.^70^ This cycle of exudation and degradation, known as sloughing, determines the thickness of the mucus layer and plays a significant role in the mucosal cleaning action.^67^ Mucus layer thickness can vary with different tissues, organ structures and under different physiological conditions.^71^ For example, within the human gastrointestinal tract, the adherent mucus gel layer in the stomach and colon is 50–200 µm deep,^72^ whilst in the eye the mucus layer is 0.2–1.0 µm.^73^ Mucus layer thickness in the colon can also be affected by microbial consortia composition, which is influenced by variability in the host diet.^74^ In healthy women, thickness and properties of cervicovaginal mucus can naturally vary throughout the menstrual cycle^75^ due to fluctuating hormones.^76^ Supplementary to these physical barriers, it is important to recognise that there are also chemical components to the defensive function of the mucus layer.

#### Physicochemical mechanisms

A key characteristic of mucin fibres, which confer the exceptional particle-trapping ability of mucus, is elasticity.^67^ Mucin fibres are flexible strings composed of alternating heavily glycosylated and hence, hydrophilic regions, with hydrophobic regions of bare protein.^67^ This conformation allows them to trap particles using myriads of low-affinity bonds that form and break easily and quickly. These intermolecular forces are also present between neighbouring mucin fibres, allowing them to re-arrange and re-anneal following shear stress.^67^ Examples of these physiochemical mechanisms can be seen in species of Cnidaria, as well as in higher organisms. For example, mucus of the beadlet anemone *Actinia equina* contains proteins that exhibit similar antibacterial activities to that of lysozyme (1,4-*β*-*N*-acetylmuramidase).^77^ This enzyme plays a vital antibacterial role by hydrolysing the peptidoglycan component of bacterial cell walls, causing cells to lyse due to increased osmotic pressure.^78^ This occurs following the breaking of glycosidic β1-4 bonds, which are present between N-acetylglucosamine (GlcNAc) and N-acetylmuramic acid (MurNAc), found in the cell walls of bacteria.^79^ The study found that the efficiency of the lysozyme-like molecule in the anemone mucus was directly affected by temperature, ionic strength and pH. The enzyme displayed optimal antimicrobial effects in test conditions that are unlikely to be experienced in the natural environment of *A. equina*, particularly the elevated temperature of 37 °C. This may indicate the limitations of this molecule in regards to its antibacterial activity at lower temperatures and higher pH values.^77^

Hard and soft corals are both capable of producing antimicrobial molecules, however their mechanism of action may vary.^80^ *Parerythropodium fulvum*, a soft coral native to the Red Sea, produces antimicrobial secondary metabolites with varying polarities. This work also provided the first evidence of antimicrobial activity in coral embryos against marine bacteria.^81^ Similarly, the Antarctic soft corals *Gersemia antarctica* and *Alcyonium paessable* are able to secrete allelochemicals such as homarine which have a bactericidal effect.^82^ These antimicrobial compounds are secreted into the mucus layer and are an important part of mucosal defence mechanisms. The efficacy of the antimicrobial compounds in the absence of mucus, or indeed the efficacy of the mucus in the absence of any additional antimicrobial compounds is difficult to measure without their chemical separation, which was not carried out in these studies.

When comparing the mucosal chemical defence of six alcyonacean soft corals and six scleractinian hard corals against *Arthrobacter* sp. and *Vibrio* sp., namely *Vibrio metschnikovii*, the mucus of alcyonacean corals showed anti-microbial activity, however the mucus of the scleractinian corals displayed little inhibitory effect.^80^ Because of this, it was concluded that only the soft coral species were able to produce anti-microbial compounds. However, the method used to identify active metabolites only identifies toxicity due to growth inhibition or cell death. Although there was no recorded effect due to production of toxins, this does not necessarily mean chemical defence mechanisms are absent within scleractinian coral mucus. For example, it has been shown that scleractinian corals have low constitutive expression of antimicrobials, but produce them rapidly upon physical damage.^83^ Furthermore, it is likely that hard corals adopt chemical defence mechanisms that are similar to those used by higher organisms, whereby metabolites are produced that target specific bacterial phenotypes.^84^ Such metabolites may affect motility, plasmid transfer, production of antibiotics and could dampen quorum sensing signals used by bacteria to mediate virulence.^85^

Metabolites employed for chemical defence within mucus have also been shown to affect fungal pathogens such as *Candida albicans*,^86^ which is associated with both superficial infection and systemic, potentially fatal diseases. *C. albicans* cells were added to media containing MUC5AC mucin and mucin-induced changes to cell morphology were monitored. The presence of mucins prevented the formation of filamentous flocs containing hyphae, which are necessary for permeation of epithelial surfaces.^87^ Instead, hyphal formation was largely suppressed and cells were only able to form short pseudohyphae. Importantly, this assay was repeated using two other mucins isolated from different sources, MUC5B mucin from human saliva and Muc2 from porcine intestinal mucus, which both showed the same effect.

Paneth cells, one of the principal, highly specialised epithelial cell types present in the mammalian small intestine,^88^ are essential for mucosal defence and maintenance of the gastrointestinal barrier. They provide protection for neighbouring stem cells, which differentiate into three other cell lineages, including mucus-secreting goblet cells.^89^ In addition, Paneth cells secrete anti-microbial proteins and peptides such as α-defensins into the mucus,^90^ creating a gradient which extends from the cell surface to the lumen.^91^ These defensins are thought to increase resistance to infection by pathogens present in the intestinal lumen.^92^ Deficiency in Paneth cell α-defensins can therefore significantly compromise mucosal immunity.^93^

Within the human colon, MUC2 mucin is responsible for the formation of polymeric nets which contain zymogen granulae protein 16.^94^ This protein is able to aggregate Gram-positive bacteria such as *Bacillus subtilis* and *Enterococcus faecalis* by binding to the peptidoglycan present in the cell wall, thus inhibiting penetration of the epithelial cells.^95^ However, Gram-negative strains investigated including *Bacteroides fragilis* and *Escherichia coli*, remained unaffected. This process allows the bacterial cells to be effectively immobilised at a safe distance from the underlying epithelium, without necessarily requiring a bactericidal effect.

The DMBT1 (deleted in malignant brain tumour 1) gene, which is expressed predominantly by epithelia in the alimentary and respiratory systems, is considered to play an important role in regulating mucosal surface homeostasis and defence.^96^ The gene encodes proteins that are involved in mucosal innate immunity, along with gp-340 (DMBT1^gp340^), a mucin-like glycoprotein and salivary agglutinin (DMBT1^SAG^).^97^ These molecules function by binding to both Gram-positive and Gram-negative bacteria and viruses.^97^ For example, DMBT1^SAG^ also binds and agglutinates oral streptococci, such as *Streptococcus mutans*.^98^ Mucus catalysed aggregation of microbial cells, therefore appears to be an important antimicrobial function.

The prevention of adherence is another method of chemical defence^42^ and may be mediated by adhesin and receptor analogues, which act as competitive inhibitors to the sites of adhesion.^99^ They function most effectively in combination with antibodies such as IgA, which is co-released in its secretory form with mucus and works by blocking the sites of adhesion and reducing motility of bacterial cells.^100^

An alternative anti-adhesive mechanism is the promotion of motility,^101^ which causes increased rates of dispersal, resulting in reduced rates of biofilm production. This was shown in *Pseudomonas aeruginosa*, where exposure to MUC5AC rich mucin significantly reduced the adhesion of *P. aeruginosa* to glass.^102^ However, the total immersion of the coverslips into the media may present a potential flaw, as realistically the bacteria would be acting at an air-liquid interface within the lungs,^103^ though there are few ways this can be replicated experimentally without specialist equipment.^104^ Conversely, mesogleal mucins from the blue blubber jellyfish *Catostylus mosaicus*, have been shown to reduce the adhesion of *P. aeruginosa* cells to human corneal epithelial cells by 86%, due to oligosaccharide attached to mucins preventing adherence by competing for binding sites upon the mucins.^42^ The oligosaccharide chains of mucins secreted by airway epithelia from cystic fibrosis patients display an abnormally high level of sulphate esters^51,105,106^ and it is suggested that sulfation might also change the binding of bacteria, although experimental evidence for this is lacking.^41^ Minimal medium-based experiments demonstrated that sulfated carbohydrates reduced the growth of *P. aeruginosa* in comparison with their non-sulfated forms,^107^ suggesting that oligosaccharide sulfation protects mucin from bacterial degradation. These results, however, need to be confirmed in more physiological environments.

**Fig. 3:**
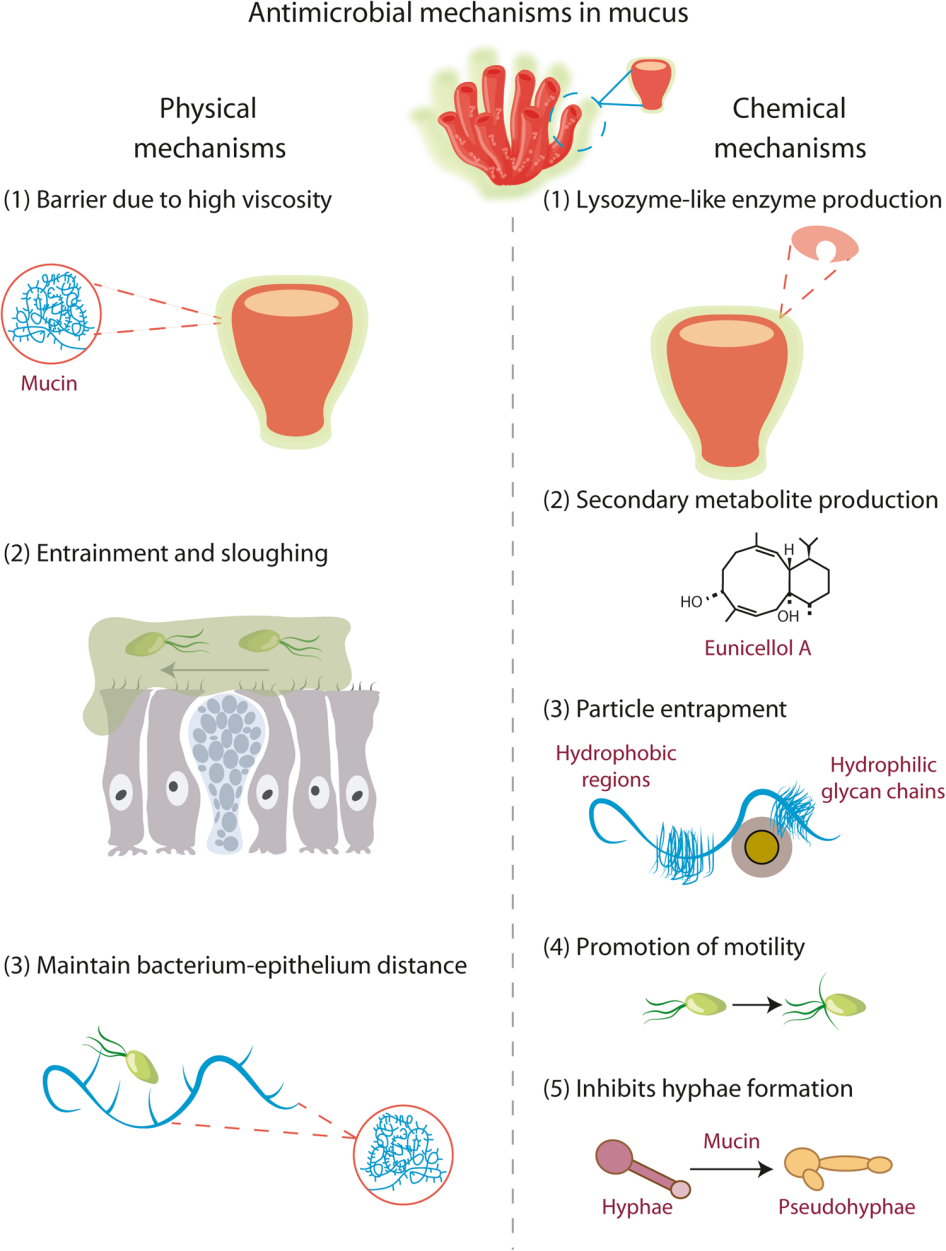
The properties of mucus, which allow resistance to microbial colonisation, can be divided into physical and chemical processes. Physical processes include its gel properties, such as thickness, entrainment, sloughing and viscosity. Chemical processes include those conferred by enzymes and secondary metabolites. The antibacterial metabolite shown here, Eunicellol A, is secreted into the mucus of the Arctic soft coral *Gersemia fruticosa*

The varied physical and chemical mechanisms of mucus used to resist colonisation and infection are presented in Fig. 3. However, despite this array of strategies, bacteria have also evolved sophisticated mechanisms to overcome them.

### Bacterial colonisation of mucus

There are two mucus layers present within the human gastrointestinal tract,^108^ one of which is adherent to the epithelial cells, whilst the other is a weaker gel which overlies this. The inner layer remains relatively devoid of microbes, whilst the outer layer contains a diverse community of commensal bacteria, necessary to ensure good health.^109^ This healthy microflora is unlikely to permeate the denser, adherent mucus, as this may stimulate an immune response, compromising this niche for the enteric microbiota.^110^ Constituent species of a healthy gut microflora, appear to have to have fewer means to allow deeper mucosal penetration, whilst pathogens are aided by increased motility and additional factors promoting attachment.^111^

Cilia-mediated mucus clearance in the airways requires an inverse composition of mucus layers. A recent 'gel-on-brush model' suggests an almost liquid mesh (also known as periciliary liquid) of membrane-spanning mucins (MUC1 and MUC4) and an overlying denser gel-like layer consisting of MUC5AC/B which serves as a particle trap.^112^ Based on the efficacy of airway muco-ciliary particle clearance, lung mucosal surfaces in healthy individuals were historically considered sterile, however, the existence of a 'lung microbiome' is recently more accepted.^113^

### Adhesion and motility

Adhesion is considered to be a critical stage in the development of an infection, contributing to the virulence of a pathogen, and where individual bacterial cells are prevented from adhering, they are considerably less likely to successfully infect the host.^114^ Bacterial adhesion may be mediated by chemical components within mucus which can act as chemoattractants.^115^ To test this, one study investigated the chemotactic response of *Vibrio anguillarum* to the skin and intestinal mucus of the rainbow trout, *Oncorhynchus mykiss*, using a chemotaxis assay, where glass capillaries containing different mucus substrates were submerged into *V. anguillarum* cultures.^116^

*Vibrio* appears to be one of the bacterial genera most able to colonise mucus in both mammals and other organisms and their virulence has been tentatively linked to their motility.^117^ *Vibrio coralliilyticus* for example, causes infection in two different species of reef-building scleractinean coral, *Pocillopora damicornis* and *Acropora millepora*.^118^ When these corals experience elevated ambient temperatures, they become susceptible to heat stress, which stimulates the release of the sulphur metabolite, dimethylsulfoniopropionate (DMSP), in high concentrations within the mucus.^118^ This elicits a chemotactic response from *V. coralliiyticus*, a highly motile bacterium that uses DMSP to target corals experiencing acute physiological stress, which are consequently most vulnerable to infection. A chemotaxis assay established that 50% of the bacterial cells present, migrated into the 400 μm thick mucus layer within 60 s of inoculation. However, one difficulty in a number of coral mucus studies, is the method used to obtain the mucus. A common method is coral milking, whereby coral specimens are partially desiccated via emersion, encouraging the production of large quantities of homogenous mucus.^60^ The mucus collected in this way has lower viscosity and is biochemically different to the mucus of the surface mucus layer.^24^ Therefore, it is likely that the anti-microbial properties of the mucus were altered significantly. Despite this, it was also found that *V. coralliiyticus* has no gene for degradation of DMSP, but the detection of the molecule stimulated chemotaxis and chemokinesis simultaneously, allowing the bacterium to substantially increase its velocity linearly with increasing DMSP concentrations, suggesting that this molecule acts solely as a potent info-chemical in this regard. DMSP itself has well-known antimicrobial properties in other systems for example it prevents grazing by ciliatea in plankton and is a widely abundant sulphur metabolite in marine ecosystems.^119^ Therefore it is probable that *V. coralliiyticus* evolved an ability to detect DMSP purely to identify heat-stressed corals, indeed DMSP has been identified as a foraging cue for other species of heterotrophic marine bacteria.^120^

Intriguingly, a similar sequence of events has been shown to occur in the cystic fibrosis lung, whereby *Pseudomonas aeruginosa*, a highly motile nosocomial pathogen, is able to target respiratory epithelial cells.^121^ When the mucociliary clearance mechanisms are impaired, an inflammatory response of the epithelial cells is to produce CXC-chemokines, which act as chemoattractants for *P. aeruginosa*.^122^ A recent in vitro study, using the human colorectal adenocarcinoma cell line Caco-2, demonstrated that unidentified low molecular weight proteins present in the supernatant of Caco-2 cultures, also attracted *P. aeruginosa* in capillary chemotaxis assays and increased bacterial motility and mucin penetration (bovine submaxillary mucin).^123^ Thus, there is growing evidence to suggest there is a link between production of chemoattractant molecules, motility of pathogens and their ability to colonise mucosal layers.

Pathogens are also able to synthesise adhesin proteins that recognise specific molecules in the mucus of the host.^68^ The strain *Vibrio* AK-1 and its attachment to the mucus of *Oculina patagonica*, a reef-building coral native to the Mediterranean, was explored using a bleaching experiment and subsequent tests using sepharose beads.^124^ It was found that coral fragments incubated at 29 °C showed 50% bleaching, whilst fragments incubated at 23 and 16 °C showed 17 and 0% bleaching, respectively. Furthermore, β-D-galactopyranoside coated Sepharose beads were then exposed to *Vibrio* AK-1 cells, and showed 98% cell adherence. These results suggested that *Vibrio* AK-1 identifies β-galactopyranosides on the coral surface using a temperature-dependent adhesin protein, which is produced at temperatures of 25 °C or above. Interestingly this correlates to the temperature range within which *O. patagonica* is likely to begin experiencing physiological stress due to elevated temperatures.^125^

### Production of mucinases

**Fig. 4:**
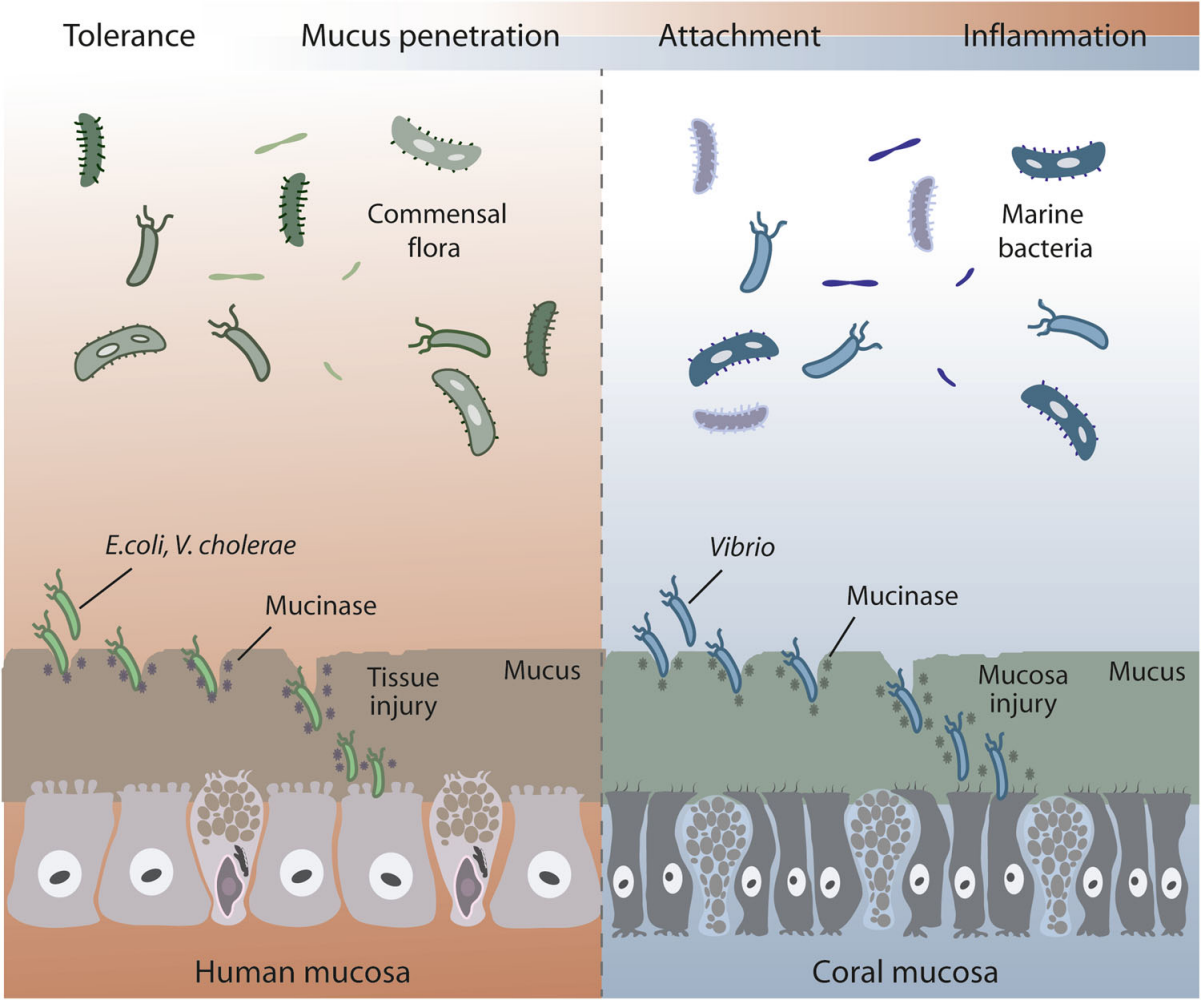
Mucinases are important hydrolytic enzymes that can contribute to the penetration of mucus, an important mechanism of bacterial pathogenesis. *E. coli* degrades mucins using a zinc metalloprotease and members of the genus *Vibrio* produce a hemagglutinin protease. Using these mucolytic enzymes, pathogens can cross human and coral protective mucus layers. Image adapted from.^135^

Another factor contributing to the pathogenicity of a bacterium is production of hydrolytic enzymes.^111^ Those that are able to penetrate mucus have the ability to produce mucinase enzymes^126^ (Fig. 4), which degrade mucin glycoproteins by breaking down oligosaccharide chains.^127^ *Helicobacter pylori*, a causal agent of gastric and duodenal ulcers in humans, has genes coding for mucinase-like enzymes. However, whilst it is possible, there is no solid evidence that *H. pylori* either produces or requires a protease to penetrate mucus. Instead it uses multiple flagella and its saw-tooth shape to bore through the mucus.^128^ In addition, once at the mucosal surface it can solubilise mucus locally, by using urease to generate a high pH which is mucolytic.^129^

When sputum from cystic fibrosis patients with a chronic *Pseudomonas aeruginosa* infection is incubated for 6 h at 37 °C, MUC5AC and MUC5B content decreases by more than 85% during incubation, an effect which was sensitive to serine protease inhibitors.^130^ There are multiple sources of proteases in cystic fibrosis airway surface liquid, including neutrophils,^131^ however, *P. aeruginosa* also releases the serine protease IV.^132^ Furthermore, the *P. aeruginosa* metalloprotease elastase B (pseudolysin) degraded both MUC5AC and MUC5B.^130^ A recent genome-wide expression screen using RNA-Seq analyses of the *P. aeruginosa* laboratory strain PAO1, identified genes such as PA3247 which code for putative proteases and are associated with the ability to breakdown mucin.^133^ As mentioned above, sulfated mucin oligosaccharides protect mucin molecules from bacterial degradation; however, bacteria including *P. aeruginosa* have evolved the ability to secrete mucin sulfatases, thus bypassing the protective chemical oligosaccharide-chain modification and enabling further proteolytic digestion of mucins.^134^

*Escherichia coli* which can cause intestinal disease, urinary tract infections and sepsis in humans,^135^ is able to cross both the outer and inner mucosal layers of the human gut epithelium via the expression of proteases^136^ such as SslE, a zinc-metalloprotease enzyme^135^ which degrades both MUC2 and MUC3 mucins expressed in adenocarcinoma cell lines.^137,138^ However, they are less glycosylated due to lower activity of glycosyltransferases^139^ and this would make them more susceptible to proteolysis than native mucins. Similarly, *Vibrio cholerae*, possesses a HapA gene encoding a soluble hemagglutinin/protease enzyme.^140^ There is some evidence to suggest that *V. cholerae* is dependent on this mucinase for its virulence, as mutants deficient in HapA are unable to degrade ovomucin from chicken eggs.^141^

The intestine of a healthy human is colonised by up to 10^14^ bacteria, belonging to about 500 different species.^142^ Whilst pathogenic bacteria are known to produce mucolytic enzymes, there are also species within the healthy gut microflora that are able to metabolise mucin-derived compounds.^143^ Due to high rates of mucus synthesis and secretion in the human gut, there is a continual influx of nutrients, which provides a novel ecological niche and direct source of nutrition for enteric microbes that are able to degrade mucus and its derivatives.^144^ This typically occurs when there is an insufficient supply of dietary fibre^145^ such as in the colon where carbon sources are more limited.^146^

*Akkermansia muciniphila* is a resident bacterium of the human gastrointestinal tract,^146^ present in approximately 75% of the population^147^ and constitutes 3–5% of the bacterial content in healthy individuals.^148^ It is adapted to degrade host mucus as a nutrient and this may provide its only carbon and nitrogen source.^149^ *A. muciniphila* produces 61 proteins (2.8% of total proteins produced) that are implicated in the degradation of mucin, including sulfatases, proteases, glycosyl hydrolases and sialidases.^147^ When grown on mucin enriched media, 30 hydrolases involved in the degradation of mucin were significantly upregulated compared to growth on glucose enriched media.^150^ However, despite living in and deriving nutrition from the mucosal environment, *A. muciniphila* does not adhere to the mucus.^151^ Its presence in the gastrointestinal tract of humans and animals may instead fortify mucosal defence by adhering to the intestinal epithelia and subsequently strengthening the enterocyte monolayer.^151^

### Similarities between the mucus layer and bacterial biofilm EPS

Bacteria, in a manner akin to higher organisms and their mucus, also possess a hydrated polymeric layer that encases their multicellular assemblies. Biofilms are the primary form of life of bacteria, in which individual cells form a dynamic and self-regulated network, whilst attached to a surface. Attachment to a surface in the form of a biofilm can often confer protection against exogenous threats and allow nutrient accumulation.^152^ Biofilms are highly tolerant to stress,^153^ as the surrounding matrix limits the mass transfer of antimicrobial agents and because biofilm cells express different genes to those of their planktonic counterparts,^154^ they have altered metabolic processes. Natural biofilms are formed by a mixture of interacting species that occupy dynamic microenvironments. Biofilms are supported by a plethora of intertwined extra-cellular polymeric substances (i.e., the EPS matrix), which is mainly composed of polysaccharides, proteins (including glycoproteins) and eDNA. The synergy between these components is key to the success of bacterial biofilms.^155^ The biofilm matrix resembles a mucus layer in many respects. It is a highly hydrated layer that provides a scaffold in which the microorganisms are embedded. The EPS matrix protects against environmental attacks, neutralising exposure against antimicrobial agents, such as biocides and antibiotics as well as xenophobic pollutants such as heavy metals and hydrocarbons. In some cases, the EPS matrix can also provide cryo and osmoprotection.^156^ Part of the protection that the EPS layer can provide stems in its ability to anchor bacteria to surfaces; this is achieved through adhesive polymers that contribute to surface adhesion as well as bacterial co-adhesion and aggregation.^157^ The hydrated matrix is a viscoelastic fluid and as such it can withstand external forces applied in a compressive, tensile or shear mode. Bacterial biofilms thus may flow along surfaces and yet remain attached. These mechanical strategies that allow them to attach, flow and relocate to more favourable niches are key to their persistence and in their ability to cause infection. The structure and composition of the biofilm directs the perfusion of nutrients. Concentration gradients of oxygen, nitrite, nitrate, ammonia and methane as well as pH also exist throughout the biofilm, to form distinct chemical microenvironments at different depths.^158^ Thus, compositional and structural properties, dependent on the matrix viscoelastic properties, are vital in the life cycle of a biofilm.^159^

It should be noted that the matrix and bacterial cell surfaces can also incorporate glycoproteins.^160^ Bacterial glycoproteins largely fall into asparagine linked (N-linked) and serine-threonine-linked (O-linked). Although mucus is also known to have the latter type of linkage, the structure of their glycoproteins varies markedly, making a direct comparison of functions difficult. In Eukaryotes glycoproteins contain N-acetylglucosamine, fucose or N-acetylgalactosamine, whereas in bacteria they contain atypical monosaccharides such as 2,4-diacetamido-2,4,6-trideoxyhexose, N-acetylfucosamine, pseudoaminic acid and legionaminic acid. Whilst glycoproteins in mucus fulfil a crucial structural role, they serve more specialised functions in bacteria: in some organisms they form crystalline S-layers, which protect bacteria against external attacks and permeate macromolecules. Glycosylated proteins are involved in bacterial pathogenicity and are components of motile and adhesive pili and flagella as well as a variety of adhesins, enabling virulence and colonisation of the host.^161^ Given the complexity of the inter-relationships between mucus and microbes, a deeper understanding of these mechanisms could lead to the development of more effective antimicrobial processes.

### Biotechnological implications

Mucins show great potential as molecules which can assist in anti-infective therapies and in recent years significant advances have been made. For example, in inflammatory bowel disease (IBD), including Crohn’s disease and ulcerative colitis, mucus layer thickness is reduced along.^162^ As a potential non-invasive treatment, it has been proposed that mucin-derived CYS domain molecules, rich in cysteine, could be administered to the gastrointestinal tract of individuals suffering with IBD.^163^ CYS domains, depending on their number and the proximity between adjacent domains, may confer the tight/loose net properties of mucus.^163^ Delivery of a molecule containing CYS domains to the mouse gut was able to fortify the mucus barrier, increasing its thickness and making it less penetrable to inert, fluorescently-labelled particles.^163^ In addition, several further changes were observed, notably, an increase in numbers of probiotic lactobacilli.^164^ This molecule also shows potential as a contraceptive, as when delivered to the cervical mucus, the polyCYS molecule favours the formation of cross-links between mucins, resulting in a change in the mucus mesh size and making it less permeable to sperm cells.^164^ Further work is required to assess the efficacy of these molecules in reinforcing the mucus layer, to prevent infection, however this is a promising advance in the application of mucus and its constituent mucins to biomedicine.

Mucin glycoproteins in cancer show modifications, both in mucin expression and their O-glycosylation profile.^165^ For example, epithelial cancer cells and early epithelial premalignant lesions are known to express immature truncated glycans.^166^ Cancer biomarker assays usually centre around detection of changes in expression and bio-distribution of products derived from cancer cells.^165^ Therefore, new assays are being developed to detect specific cancer-associated glycoforms of mucins.^165^

Jellyfish mucins have been described as potential candidates for manufacturing protective coatings, as they are non-toxic and can be harvested from all parts of the jellyfish.^42^ In addition, it has been speculated that mucins could be used in future to coat implants.^86^ More specifically, Muc5b mucin has been proposed as a useful indicator to track the occurrence and monitor treatment efficacy in ocular diseases such as dry eye syndrome.^167^ Mucus is also being explored as a composite material in hydrogels to cover wounds and prevent nosocomial infection following surgery.^168^ However, as mucins form gels under low ionic strength and low pH, they are not compatible with the external wound environment, thus mucin hydrogels require reconstitution with other macromolecules such as methylcellulose, which act as an adjuvant to form a hybrid biopolymer. There are some limitations to the biotechnological use of mucus, which include the difficulty in harvesting this substance in large quantities without exploiting and detrimentally impacting natural populations.^169^ Additionally, acquisition of mucins from the crude mucus on a commercial scale may require volatile toxic chemicals, which could compromise their use in biomedicine.^42^ Despite this, Qniumucin has been investigated for its potential in the treatment of osteoarthritis^170^ and has since entered clinical trials to establish its suitability for use in regeneration of artificial cartilage.^171^ In addition, bioactive compounds are beginning to be isolated from catfish mucus, which could be used in wound healing, as they have an inhibitory effect on growth of clinically relevant pathogens including *Pseudomonas aeruginosa*, *Staphylococcus aureus* and *Escherichia coli*, which is similar to that of gentamicin.^172^ Progress in this area however, will rely on the development of methods that allow large yields of mucus to be obtained, and this is not yet possible.

### Experimental techniques used in the collection and study of mucus

Investigating the in situ biophysical and functional properties of mucus has been experimentally challenging due to the limited number of suitable methods that can be used to obtain and purify mucins. This is partly due to their complexity, large size and heterogeneity.^173^ Whilst they are available commercially, the industrial method of preparation usually requires treatment with proteases, which causes the mucin fibres to become un-cross-linked and degraded^174^ rendering them incapable of forming their original physiologically relevant hydrogel structure.^5,41,102,175^ Therefore, whilst it is still possible to study the composition and constituents of mucus, to fully understand its barrier properties it is essential that it is able to reanneal under experimental conditions.

Methods to remove bacteria within mucus samples such as autoclaving and filtration present additional difficulties. Autoclaving denatures proteins due to the prolonged period of intense heat and pressure, whilst filter sterilisation is entirely ineffectual due to mucus viscosity. This makes working on the microbiological components of natural mucus particularly challenging. Whilst studies are available that described the process used to obtain and remove debris from porcine stomach and small intestinal mucus,^176^ there is a paucity of detailed protocols which document reliable methods of subsequent mucin purification from crude mucus exudate in the literature. The most appropriate methods involve homogenisation, centrifugation, freeze-drying and recombination of the individual components. This process is time consuming, taking approximately one week to purify 1 g of gastric mucins and 2 weeks to purify 1 g of intestinal mucins.

## Conclusions

There is increasing evidence to indicate that mucin genes are functionally conserved throughout metazoan evolution from early evolving metazoans, such as cnidarians and ctenophores, to higher organisms, including terrestrial mammals and humans.^40,42^ Nevertheless, whilst some work is being done to reveal the evolutionary basis of these complex glycoproteins,^3,26^ the importance of the early evolution of mucus has not previously been recognised. The study of the properties of the mucus layer is a critically important area that can provide insights into their molecular structure, and the precise mechanisms by which infection can be thwarted.
